# Commercial Production of Highly Rehydrated Soy Protein Powder by the Treatment of Soy Lecithin Modification Combined with Alcalase Hydrolysis

**DOI:** 10.3390/foods13121800

**Published:** 2024-06-07

**Authors:** Shuanghe Ren, Yahui Du, Jiayu Zhang, Kuangyu Zhao, Zengwang Guo, Zhongjiang Wang

**Affiliations:** 1 College of Food Science, Northeast Agricultural University, Harbin 150030, China; ren10180132@163.com (S.R.); 19861170531@163.com (Y.D.); zjiayu0224@163.com (J.Z.); gzwname@163.com (Z.G.); 2 Fang Zheng Comprehensive Product Quality Inspection and Testing Center, Fangzheng County, Harbin 150800, China; 18686860676@163.com

**Keywords:** soy protein powder, soy lecithin, enzymatic hydrolysis, rehydration property, emulsifying property

## Abstract

The low rehydration properties of commercial soy protein powder (SPI), a major plant−based food ingredient, have limited the development of plant−based foods. The present study proposes a treatment of soy lecithin modification combined with Alcalase hydrolysis to improve the rehydration of soy protein powder, as well as other processing properties (emulsification, viscosity). The results show that the soy protein–soy lecithin complex powder, which is hydrolyzed for 30 min (SPH–SL−30), has the smallest particle size, the smallest zeta potential, the highest surface hydrophobicity, and a uniform microstructure. In addition, the value of the ratio of the α−helical structure/β−folded structure was the smallest in the SPH–SL−30. After measuring the rehydration properties, emulsification properties, and viscosity, it was found that the SPH–SL−30 has the shortest wetting time of 3.04 min, the shortest dispersion time of 12.29 s, the highest solubility of 93.17%, the highest emulsifying activity of 32.42 m^2^/g, the highest emulsifying stability of 98.33 min, and the lowest viscosity of 0.98 pa.s. This indicates that the treatment of soy lecithin modification combined with Alcalase hydrolysis destroys the structure of soy protein, changes its physicochemical properties, and improves its functional properties. In this study, soy protein was modified by the treatment of soy lecithin modification combined with Alcalase hydrolysis to improve the processing characteristics of soy protein powders and to provide a theoretical basis for its high−value utilization in the plant−based food field.

## 1. Introduction

As concerns about the sustainability of animal protein in diets have increased, consumer preferences have gradually shifted to diets rich in plant proteins [1]. The surge in global demand for alternative protein sources in recent years, particularly for the development of new plant−based food products, has highlighted soy as a prominent plant protein option for vegan and vegetarian diets [2]. In addition to its nutritional benefits, soy protein possesses emulsification, gelation, foaming, and other functional properties [3,4]. Soy protein in the food industry is commonly spray−dried into powder to enhance shelf life and decrease expenses related to storage and transportation. However, the inadequate rehydration of soy protein powder resulting from spray drying is a significant limitation due to the inherent characteristics of the raw material and processing parameters [5]. This limitation significantly hinders the utilization of soy protein powder in plant−based food products, particularly in plant−based beverages [6]. Poor rehydration of soy protein powder not only seriously interferes with the operation of the wetting unit at the industrial level, but also detrimentally impacts the functional properties, including emulsification, gelation, and foaming, of the protein [7]. For the plant−based beverage industry, soy protein powders with poor rehydration properties can directly affect the stability of the beverage system, leading to sedimentation and rough taste, thereby reducing the product quality [8]. Therefore, the plant−based food industry, especially the plant−based beverage industry, has an urgent need for soy protein bases with good rehydration properties.

In recent years, various physical, chemical, and enzymatic methods have been employed to enhance the rehydration of protein powders. Wu et al. [9] utilized polysorbate 80, a surfactant, to enhance the rehydration of milk protein isolate (MPI) powders through surface property modifications. Wang et al. [8] pointed out that ultrasound pretreatment (UP) can improve the rehydration of egg white powder by mitigating interactions among high−abundance proteins and increasing their solubility. Tang et al. [7] showed that subtilisin significantly improved the solubility of egg yolk powder (EYP), potentially attributed to the decreased surface hydrophobicity of EYP following enzymatic hydrolysis. It is evident that previous investigations have primarily concentrated on enhancing the rehydration characteristics of MPI and EYP powders, with a noticeable lack of emphasis on improving the rehydration properties of SPI powders. This deficiency in research hinders the advancement of plant−based food matrices. Soy lecithin (SL), as a natural mixed surfactant, possesses physiological benefits including blood lipid regulation, cognitive enhancement, and anti−aging properties [10]. The asymmetric structure of hydrophobic alkyl side chains and hydrophilic groups in SL alters the surface properties of protein powder, leading to enhanced rehydration properties [11]. Therefore, SL has been utilized to enhance the rehydration characteristics of soy protein powders. However, too much SL can easily cause a series of quality problems, such as deterioration of protein powder flavor and uneven dispersion of lecithin [12]. A small amount of SL improves protein powders weakly. Therefore, new processing methods are needed to improve the rehydration of commercially produced SPI powders. Alcalase enzymatic hydrolysis is an effective and efficient means of increasing protein solubility [13]. Alkaline proteases can alter the solubility of proteins by affecting the molecular weight, hydrophobicity, and spatial structure of protein hydrolysates, as well as ionizable and polar groups [14]. Though enzymatic hydrolysis results in increased protein solubility, the enzymatic product will often show poor emulsification properties due to the imbalance between hydrophilic and hydrophobic groups [15]. This is also not beneficial to the processing of plant−based foods. The treatment of soy lecithin modification combined with Alcalase hydrolysis may be able to achieve a balance between the hydrophilic and hydrophobic groups of proteins and more effectively improve the rehydration, emulsification, and other processing qualities of commercial protein powders. However, the treatment of soy lecithin modification combined with Alcalase hydrolysis to improve rehydration of commercially produced soy protein powders is less studied.

Therefore, soy protein isolate powder (SPI), soy protein isolate−soy lecithin complex powder (SPI−SL), and soy protein isolate−soy lecithin complex powder treated with different Alcalase hydrolysis times (SPH−SL) were selected for study. The impact of different Alcalase hydrolysis times on the rehydration characteristics of soy lecithin−soy protein isolate complex powders are discussed, establishing correlations between physical attributes, protein structure, and rehydration properties. These findings offer a theoretical framework for enhancing the rehydration properties of soy protein powders.

## 2. Materials and Methods

### 2.1. Materials

Defatted soybean meal was procured from Shandong Yuwang Ecological Food Industry Company Incomplete (Dezhou, China). Alcalase (200 U/mg) was purchased from Solarbio Science & Technology Co., Ltd., Beijing, China, while soy lecithin was sourced from Yuanye Biotechnology Co., Ltd., Shanghai, China. All additional reagents utilized in this research were of analytical grade, and were also procured from Yuanye Biotechnology Co., Ltd., Shanghai, China.

### 2.2. Sample Preparations

#### 2.2.1. Preparation of SPI

The SPI extraction procedure was conducted following the protocol outlined by Lian et al. [16], with minor adjustments. Defatted soy flour was dispersed in deionized water (1:10, *w*/*v*), and the pH of the dispersion was regulated to 8.0 using 2M NaOH. The mixture was subjected to centrifugation (4 °C, 8000× *g*, 30 min) after being stirred for 2 h at room temperature, and the resulting supernatant was harvested. The pH of the supernatant was adjusted to 4.5 with 2 M HCl then allowed to stand overnight at 4 °C. Following another round of centrifugation (4 °C, 8000× *g*, 30 min), the precipitate was washed three times with distilled water. The precipitate was subsequently redispersed in distilled water to create a concentrated stock solution of soy protein. The pH of the soy protein stock solution was neutralized to 7.0 using a 2 M NaOH solution, followed by the production of SPI through homogenization at 20 MPa and subsequent spray drying. Determined by the Kjeldahl method, the protein content of the extracted SPI is 91.6%.

#### 2.2.2. Preparation of SPI–SL

SL was incorporated into SPI stock solution at a concentration ratio of 1:10. The pH of the resulting solution was subsequently adjusted to 7.0 using 2 M NaOH to create the SPI–SL stock solution. This stock solution was then subjected to homogenization at 20 MPa and subsequently spray−dried to produce the final SPI–SL product.

#### 2.2.3. Preparation of SPH–SL

The stock solution of SPI–SL was processed by enzymatic hydrolysis using alkaline protease (55 °C, pH:7.0). The pH of the enzymatic hydrolysate was regulated with 2 M NaOH, with the consumption of NaOH being monitored. Following a specified duration of enzymatic hydrolysis, the enzymatic hydrolysate was subjected to heat treatment at 95 °C for 10 min and subsequently cooled in an ice bath to halt the hydrolysis process. The solution after enzymatic hydrolysis was homogenized by a high−pressure homogenizer (Dyhydromatics, Maynard, MA, USA) at 20 MPa, and then sprayed to obtain SPI–SL hydrolysates (SPH–SL). Taking SPH–SL−5 as an example, it represents the protein powder obtained by SPI–SL enzymatic hydrolysis for 5 min.

### 2.3. Determination of the Degree of Hydrolysis (DH)

In accordance with the methodology outlined by Hao et al. [17], a slight modification was made to determine the degree of hydrolysis (DH). Throughout the enzymatic process, 0.1 mol/L NaOH was consistently introduced to sustain the optimal pH of 7 in the enzymatic hydrolysate, with the quantity of NaOH utilized being documented. The DH was calculated with Formulas (1) and (2):

(1) $$\begin{array}{c} DH\left(\%\right)=\frac{N_{b}\times V\times 100}{\alpha \times M_{p}\times h_{tot}} \end{array}$$

(2) $$\begin{array}{c} \alpha =\frac{10^{pH-pK}}{1+10^{pH-pK}} \end{array}$$

where *V* NaOH represents the quantity of alkali consumed (mL), *N* NaOH denotes the molarity of alkali (mol/L), α signifies the calibration factor for pH−stat, which is contingent upon pH, *M_p_* indicates the mass of the SPI (g), and *h_tot_* signifies the total number of peptide bonds; that is, 7.78 mmol/g protein.

### 2.4. Average Particle Size and Zeta Potential

The powder samples were prepared as a 0.1% protein solution in deionized water and subjected to particle size and zeta potential analysis at room temperature using the zeta Size Nano ZS instrument (Malvern Instruments, Worcestershire, UK). Each measurement was conducted in triplicate.

### 2.5. Turbidity

Each sample was dispersed in deionized water to achieve a concentration of 1.0 mg/mL protein solution [18]. Subsequently, the turbidity of all samples was assessed by measuring the absorbance at 600 nm using a UVmini−1240 spectrophotometer (Shimadzu Research Laboratory (Shanghai) Co. Ltd., Shanghai, China).

### 2.6. Scanning Electron Microscopy

All powder samples were affixed to double−sided adhesive tape on an aluminum column. The microstructure of the powders was observed by using a tungsten scanning electron microscope (JSM−6390LV, JEOL, Tokyo, Japan) at an accelerating voltage of 15 kV.

### 2.7. FTIR Analysis

The Fourier transform infrared spectra of the specimen were analyzed using an FTIR spectrophotometer (Nicolet Bruker iS10 spectrophotometer, Thermo Scientific, Waltham, MA, USA) within the wavenumber range of 4000 to 500 cm^−1^. Detailed information regarding the secondary structure of the samples was obtained through Fourier self−deconvolution within the amide I region (1700–1600 cm^−1^) utilizing PeakFit Version 4.12 software (SPSS, Inc., Chicago, IL, USA). The absorption peaks resulting from C=O stretching vibration in the amide I spectrum indicate different secondary structures of proteins, including α−helix: 1650–1660 cm^−1^; β−sheet: 1610–1640 cm^−1^; β−turn: 1661–1700 cm^−1^; random coil: 1640–1650 cm^−1^ [19]. Each sample was measured in triplicate.

### 2.8. Surface Hydrophobicity

The samples were prepared through phosphate buffer to obtain concentrations of 0.02–0.1 mg/mL. Then, 50 μL ANS (8 mmol/L) and 4 mL protein solution was mixed by incubating at room temperature for 30 min. The fluorescence intensity was measured through Hitachi F−7000 fluorescence spectrometer (Hitachi, Tokyo, Japan). Excitation and emission wavelength were 390 nm and 470 nm respectively, the scanning slit was 10 nm, and the scanning speed was 10 nm/s. Linear regression was used to fit the plot of fluorescence intensity vs. protein concentration. The slope of the curve was used to define the H0 of samples.

### 2.9. Bulk Density

The bulk density of the powder sample was determined by dividing the mass of the sample by its volume [20]. The methodology employed in this study was adapted from the approach outlined by Senka S. Vidović et al. [21], with simple modifications. Specifically, a 15 g powder sample was carefully placed into a 100 mL graduated cylinder, which was then subjected to vibration for 3 min to ensure accurate volume measurement. Each sample was analyzed in triplicate to ensure precision. The bulk density of the sample can be calculated using Formula (3):

(3) $$\begin{array}{c} Bulk\;density\left(\%\right)=\frac{m}{v}\times 100\% \end{array}$$

where *m* is the weight of sample, *v* is volume of sample.

### 2.10. Rehydration Properties

#### 2.10.1. Wettability

The wettability of the powder samples was assessed through dispersion time, following the protocol outlined by Gong et al. [22]. Each powder sample (1 g) was introduced into a beaker with 40 mL of water and subjected to magnetic stirring at 300 rpm/min at room temperature. The time taken for complete wetting, from the initiation of stirring, was documented as the wettability time for powder evaluation. Powders achieving wetness within 60 s were classified as easily wettable, whereas those surpassing 120 s were deemed non−wettable.

#### 2.10.2. Dispersibility

As per the methodology outlined by Felix da Silva, Ahrné et al. [23], the dispersibility of the powder samples was assessed by the duration required for complete moistening and dispersion of the powder agglomerates upon initial agitation. This assessment was repeated three times to determine the dispersibility of all powder samples, with the average dispersion time being calculated.

#### 2.10.3. Solubility

Following the methodology outlined by Tan et al. [24], with minor adjustments, the solubility of the samples was assessed. Each sample (0.2 g) was dissolved in 5 mL of deionized water and agitated at ambient temperature for 60 min, then centrifuged at 10,000× *g* for 20 min. The protein content of the supernatant was quantified using the bicinchoninic acid (BCA) protein detection kit (Beyotime Biotechnology Co., Ltd., Shanghai, China), with bovine serum albumin employed as the protein standard. To be specific, the 20 μL sample and 200 μL BCA working liquid were placed in the sample hole of the 96−well plate at 37 °C for 30 min. The absorbance of protein at 562 nm was measured by microplate readers (INFINITE M200 PRO), and the protein concentration of the sample was calculated reference to the standard curve.

### 2.11. Viscosity

The viscosity of all sample solutions was measured using a rotational viscometer (LV—DVS+, Brookfield Engineering Laboratories Inc., Middleboro, MA, USA). In the measurement of viscosity, the rotor type is LV−1 and the shear rate is 60 rpm. The sample solution for the viscosity measurement had a protein concentration of 10%.

### 2.12. Emulsify Property

Emulsifying property is indicated by the emulsion activation index and emulsion stability index (EAI and ESI). The method for evaluating these properties was based on the protocol developed by Kinsella and Pearce [25], with minor adjustments. Specifically, soy oil was mixed with the SPI sample solutions at a concentration of 1 mg/mL in a 1:3 (*v*/*v*) ratio, followed by homogenization using a homogenizer (ANGNI Co. Ltd., Shanghai, China) at 11,000 rpm for 2 min. Emulsions were sampled at 10 min and 0 min, and 50 μL of each sample was diluted 100 times with a 0.10% (*w*/*v*) solution of sodium dodecyl sulfate (SDS). The absorbance of the emulsion at 500 nm was recorded using a spectrophotometer (SHJH Co. Ltd., Shanghai, China). EAI (m^2^/g) and ESI (%) were evaluated according to Equations (4) and (5), respectively.

The emulsifying activity index (EAI) and emulsion stability index (ESI) were computed using the respective equations.

(4) $$\begin{array}{c} EAI\left(\frac{m^{2}}{g}\right)=\frac{2\times T\times DF\times A_{0}}{\left(1-\theta \right)\times C\times L\times 1000} \end{array}$$

(5) $$\begin{array}{c} ESI\left(\%\right)=\frac{A_{10}}{A_{0}}\times 100\% \end{array}$$

where *A*_0_ is the absorbance at 0 min, *A*_10_ is the absorbance at 0 min, *D* is the dilution factor (100), *φ* is the oil phase volume fraction of the emulsion (*v*/*v*) (0.25), *C* is the concentration of protein solution before emulsification (mg/mL), *T* is the turbidity (2.303).

### 2.13. Statistical Analysis

The experiments were conducted in triplicate, and the results were presented as the mean values accompanied by standard deviations. The data collected were subjected to statistical analysis through a one−way analysis of variance (ANOVA) and subsequently Duncan’s test, utilizing SPSS 26 software. A significance level of *p* < 0.05 was employed to determine statistical significance.

## 3. Results

### 3.1. The DH of SPH−SL at Different Hydrolysis Times

**Figure 1:**
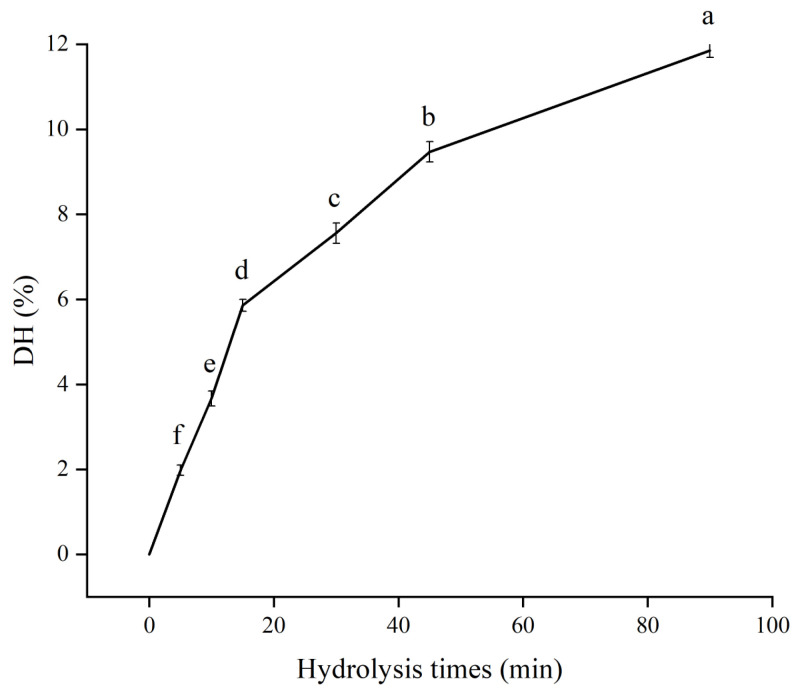
The DH of SPH−SL at different hydrolysis times. The different superscript letters indicate significant differences (*p* < 0.05).

The DH value reflects the degree of cleavage of protein peptide bonds during hydrolysis, impacting the size and amino acid composition of the peptide and subsequently influencing the protein’s interaction with SL [26]. It will improve the functional properties of the protein. Figure 1 shows the DH of SPH−SL at different hydrolysis times. The figure shows a direct correlation between the DH values of SPH−SL and the duration of hydrolysis, as the DH of SPH−SL increased with the increase in the hydrolysis times. It is associated with extensive peptide bond cleavage catalyzed by Alcalase, which may mostly occur on the surface of soy protein aggregates [27]. This means that as the enzymatic hydrolysis time increases, the amount of large molecule proteins will decrease and the amount of small molecule peptides or amino acids will increase. The formation of small molecule short−chain substances may contribute to the rehydration properties of soy proteins [28]. K. Surówka further highlighted that enzymatic hydrolysis disrupts the native molecular configuration, leading to the exposure of hydrophobic and hydrophilic regions on the protein surface, with the hydrophilic regions facilitating the protein’s interaction with water [29]. In addition, the hydrophilic head or hydrophobic long chain of lecithin may bind to the hydrophilic and hydrophobic group sites exposed by enzymatic hydrolysis of soy protein isolate, leading to changes in particle size and surface properties of protein molecule [30]. This will further affect the rehydration properties of the protein.

### 3.2. The Particle Size of SPH−SL at Different Hydrolysis Times

**Figure 2:**
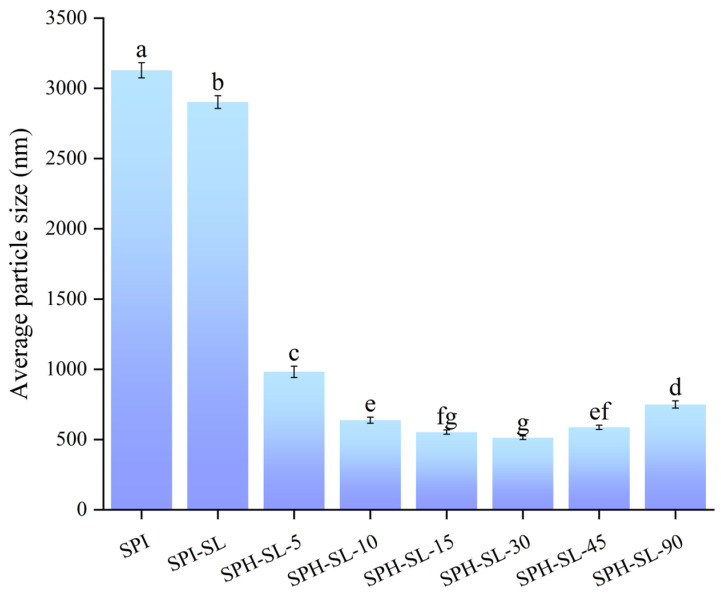
The average particle size of SPH−SL at different hydrolysis times. The different superscript letters indicate significant differences (*p* < 0.05).

The particle size of proteins can reflect the contact area of proteins with water and the interaction between proteins and water, which will significantly affect the rehydration properties (wettability, solubility, and dispersibility) of proteins [22]. The average particle size of SPH−SL at different hydrolysis times is shown in Figure 2.

Figure 2 found that the SPI–SL has a lower average particle size than SPI. The alteration in protein particle size resulting from soy lecithin complexation treatment may be attributed to the change of protein surface activity and charge by SL, leading to an increase in repulsive forces between protein particles. Additionally, the average particle size of all SPH−SL samples was found to be smaller than that of SPI−SL and SPI. Moreover, the average particle size of SPH−SL decreased initially and then increased as the hydrolysis time increased. Notably, the average particle size of SPH−SL−30 was the smallest among the samples studied. These findings, in conjunction with the particle size data, suggest the treatment of soy lecithin modification combined with Alcalase hydrolysis has a better effect in reducing the particle size of soy protein compared to the single soy lecithin modification treatment. The effective reduction of protein particle size by the treatment of soy lecithin modification combined with Alcalase hydrolysis may be attributed to the increased exposure of binding sites on proteins following enzymatic hydrolysis. This exposure facilitates the complexation of soy lecithin and soy protein, leading to enhanced repulsion between protein particles. However, the particle size of the SPH–SL tends to increase with prolonged enzymatic hydrolysis, potentially due to the aggregation of peptides and amino acids resulting from hydrophobic interactions during enzymatic hydrolysis. The small particle size exhibited by SPH−SL suggests that it may have good solubility. A smaller average particle size of SPH−SL results in a higher surface−to−volume ratio, leading to greater separation pressure for the colloidal particles and promoting the dispersion of the protein. This separation pressure is determined by the attractive force between water and particles divided by their area [31]. Moreover, a reduction in the average particle size of SPH–SL enhances the probability of protein–water interaction, consequently enhancing the solubility of the protein [32].

### 3.3. The Turbidity of SPH−SL at Different Hydrolysis Times

**Figure 3:**
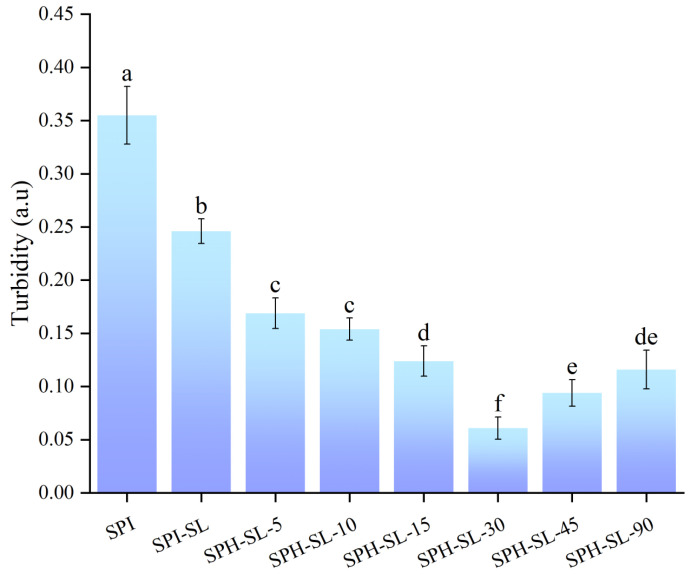
The turbidity of SPH−SL at different hydrolysis times. The different superscript letters indicate significant differences (*p* < 0.05).

Turbidity, defined as the extent to which suspended particles in a solvent obstruct light scattering or dispersion, serves as a measure of solution transparency and the aggregation or dispersion of protein particles [33]. It significantly impacts the sensory properties and protein rehydration characteristics of protein solutions. Generally, turbidity is correlated with the size and concentration of colloidal particles in a protein suspension [34]. The turbidity of SPH−SL at different hydrolysis times is shown in Figure 3.

Figure 3 found that the turbidity of SPH−SL was less than that of SPI−SL. The turbidity of SPI−SL was lower than that of SPI. The turbidity of SPH–SL decreased and then increased with increasing hydrolysis time, and the turbidity of SPH−SL−30 was the lowest. The lower turbidity of SPH−SL indicates that SPH−SL has good dispersion and a clear appearance. In addition, turbidity generally has an opposite trend to solubility [18]. Therefore, SPH−SL with low turbidity may also have good solubility. The change in turbidity of all samples is consistent with the change in particle size. This may be related to the low light dispersion and light scattering of proteins with small particles [35]. In addition, the change in turbidity of all samples could also be related to soy lecithin modification and Alcalase hydrolysis treatment altering the surface charge or structure of the proteins, leading to protein aggregation or dispersion. This will be explored in the next analyses. In conclusion, the treatment of soy lecithin modification combined with Alcalase hydrolysis can effectively improve turbidity, and enhance water dispersibility and the appearance of commercial soy isolated proteins for processing applications.

### 3.4. The Zeta of SPH−SL at Different Hydrolysis Times

**Figure 4:**
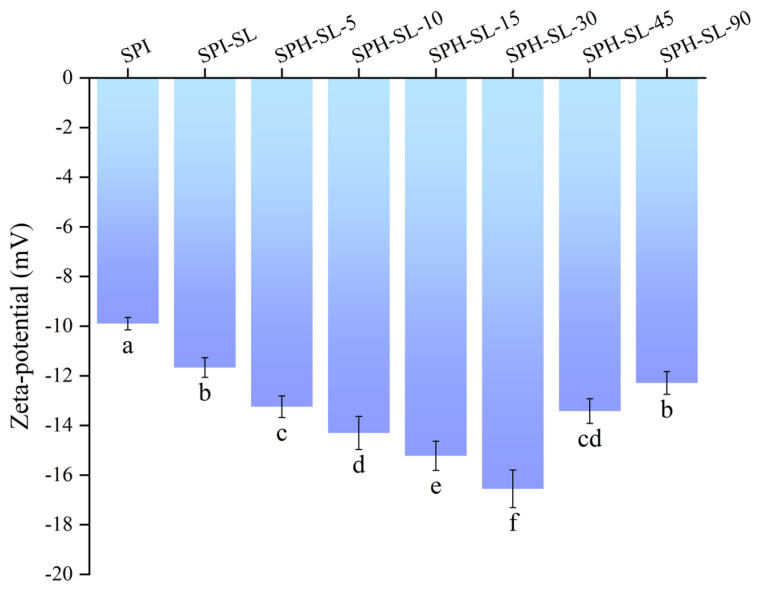
The zeta potential of SPH−SL at different hydrolysis times. The different superscript letters indicate significant differences (*p* < 0.05).

The zeta potential of a protein is indicative of its surface charge and offers valuable information regarding the electrostatic interactions among protein particles. This property plays a significant role in influencing the rehydration properties and aggregation behavior of proteins [36]. Normally, a higher absolute value of the negative ζ−potential signifies higher net negative surface charges and greater electrostatic repulsion, which is favorable for enhancing system stability [37]. The zeta potential of SPH–SL at different hydrolysis times is shown in Figure 4.

Figure 4 shows that the zeta potential of SPI is negative. The potential of SPI–SL is less than that of SPI, which may be because SL has a negatively charged phosphate group [38]. The potential of SPH−SL decreased and then increased with increasing hydrolysis time, and the potential of SPH–SL−30 was the smallest. This may be due to the conformation of the soy protein changing during a short period of hydrolysis, exposing more polar groups. This may facilitate the formation of complexes between soy protein and SL, leading to an increase in the quantity of charged groups. However, as the enzymatic hydrolysis time is too long, aggregation between proteins may occur. The aggregated SPH−SL may rehide the exposed surface polar groups inside the protein, which may result in a reduction in the number of ionizable groups of the protein. In addition, the zeta potential of all SPH−SL was smaller than that of SPI−SL and SPI. It means that treatment of soy lecithin modification combined with Alcalase hydrolysis can significantly improve the zeta potential of proteins. The smaller zeta potential of SPH−SL means that SPH−SL has more negative charge and higher electrostatic repulsion. This may help to increase the separation distance between protein particles and prevent agglomeration of protein particles, thus enhancing system stability [39]. Regarding this, the phenomenon of zeta potential is consistent with that of particle size and turbidity. Furthermore, the more negative charge of SPH−SL could mean that SPH−SL would have stronger protein−water interactions, thereby improving solubility [31].

### 3.5. The Morphology of SPH−SL at Different Hydrolysis Times

**Figure 5:**
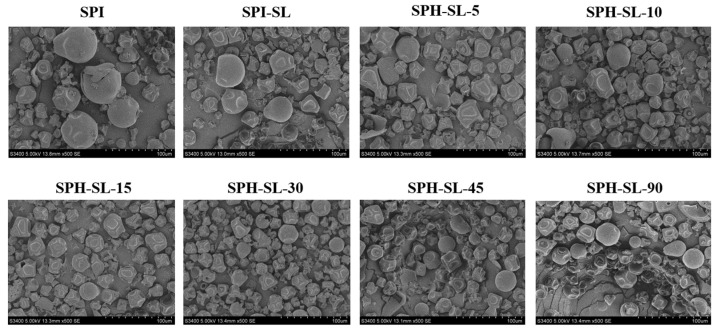
The morphology of SPH−SL at different hydrolysis times.

The morphology of the particles, which significantly influences the functional characters of soy protein, was characterized by SEM. Figure 5 illustrates the morphology of SPH–SL at different hydrolysis times.

It can be observed from Figure 5 that all samples exhibit a similar morphology, with the majority of particles displaying a collapsed structure, while a minority appear round and smooth. This can be attributed to the high air temperature during spray drying. Notably, there are notable variations in particle size and distribution among different samples. In comparison to SPI, SPI−SL demonstrates a reduction in particle size and a more uniform distribution, attributed to alterations in protein surface composition induced by soy lecithin. This leads to a decrease in protein aggregation. The particle size of SPH−SL initially decreases and then increases with prolonged hydrolysis time, while its distribution transitions from uniform to aggregated. Among the samples tested, SPI–SL−30 exhibits the smallest particle size and most uniform distribution, aligning with the average particle size results. It may be related to the Alcalase hydrolysis promoting the complexation of SL and soy protein, increasing electrostatic repulsion and disrupting hydrophobic interactions, thus reducing protein aggregation [40]. In contrast, prolonged enzymatic hydrolysis may expose hydrophobic groups on proteins, peptides, and amino acids (enzymatic hydrolysates), promote aggregation of enzymatic hydrolysates, and lead to larger particle size and uneven distribution of SPH−SL [41]. During the dispersion process, the uniform and small particle morphology of SPH–SL may create more opportunities for proteins to come into contact with water molecules, thus altering the rehydration properties of proteins [32].

### 3.6. The FTIR Spectra of SPH−SL at Different Hydrolysis Times

**Figure 6:**
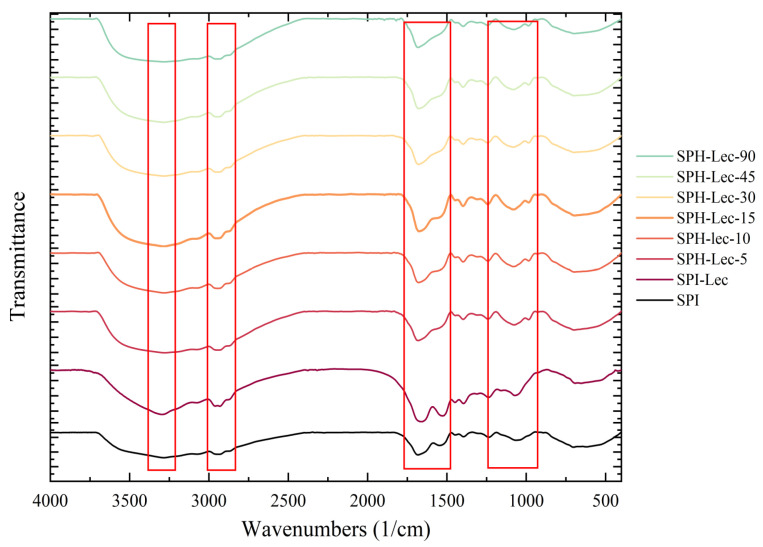
The FTIR spectra of SPH−SL at different hydrolysis times.

FTIR spectra were utilized to analyze the alterations in vibrational frequencies of functional groups and secondary structure within soy protein [42]. The FTIR spectra of SPH–SL were examined in the 4000−400 cm^−1^ range at various hydrolysis durations, as illustrated in Figure 6.

For instance, in the case of SPI, the broad bands observed in the 3200−3400 cm^−1^ region were attributed to intermolecular N−H and O−H stretching vibrations of the protein, indicating the presence of hydrogen bonding [43]. The peaks at 2935 cm^−1^ in the SPI spectrum were attributed to asymmetric stretching vibrations of the CH_2_ group in the aliphatic side chain, reflecting the hydrophobic region of the protein structure [44]. The peaks at 1655 cm^−1^and 1540 cm^−1^ in the SPI spectrum are attributed to amide I and amide II of protein, respectively, which represents the typical characteristics of protein conformation and structure [45].

No new absorption peaks were observed to appear in SPI−SL compared to SPI, while some minor characteristic absorption peaks were changed in SPI−SL between 1500 cm^−1^ and 500 cm^−1^, which is related to the P = O bonding of SL. This implies some weak physical interaction between SL and soy protein. Compared to SPI, the hydroxyl peak in SPI−SL shifted to the high wavenumber region, the peak of CH_2_ group stretching vibration in SPI−SL shifted to the low wavenumber region, and the amide I and II bands in SPI−SL were significantly altered. This may be attributed to the asymmetric structure of the hydrophobic alkyl side chains and hydrophilic groups of SL, as well as the binding of SL to soy proteins through electrostatic, hydrophobic, and hydrogen bonding interactions [46]. This would potentially improve the soy protein structure and increase the hydrophilicity of soy proteins. In contrast to SPI and SPI−SL, the hydroxyl peaks of SPH−SL were shifted to lower waves, while the CH_2_ group stretching vibration peaks of SPH−SL were shifted to higher waves. This may mean that enzymatic treatment further disrupted the intermolecular hydrogen bond structure and the hydrophobic structural domain of SPI. However, the hydroxyl and the CH_2_ group stretching vibration peaks of SPH−SL did not change significantly with increasing hydrolysis time. Furthermore, it is also noteworthy that the amide I and amide II bands of SPH−SL gradually weakened with increasing hydrolysis time. This may be attributed to the fact that Alcalase hydrolysis treatment disrupts the backbone structure of soy protein and facilitates the unfolding of the protein structure [47]. Therefore, the treatment of soy lecithin modification combined with Alcalase hydrolysis significantly altered the structure and the hydrophobic structural domain of soy protein.

To understand the structural changes of SPH−SL at different hydrolysis times, amide I bands of all samples were fitted and the results are shown in Table 1. The ratio of α−helix and β−folded structures can reflect the protein flexibility, which affects solubility, as has been documented in the literature [48]. A lower ratio of α−helix to β−folded structures indicates greater protein flexibility and enhanced interaction with water molecules, leading to increased solubility [49]. Compared with SPI, the α−helix proportion and β−turn proportion of SPI−SL decreased, and the β−sheet proportion and random coil proportion of SPI−SL increased. This may be due to SL combining with the hydrophobic groups of soy protein, leading to the rupture of its hydrogen bonding network [50]. The α−helix/β−sheet ratio of SPI−SL is also lower than the α−helix/β−sheet ratio of SPI, which implies that SPI–SL may have a more flexible structure than SPI, thus improving protein solubility. In the case of SPH–SL, as hydrolysis time increased, the proportion of α−helix decreased initially, then increased, while the proportion of β−sheet increased initially, then decreased. Additionally, the proportion of β−turn increased steadily, while the proportion of random coil decreased consistently. Concurrently, the ratio value of α−helix/β−sheet of SPH–SL increased and then decreased with increasing hydrolysis time. Among all samples, SPH–SL−30 has the smallest α−helix structure and the highest β−sheet structure, resulting in the smallest α−helix/β−sheet ratio of SPH–SL−30. This implies that SPH–SL−30 has a flexible structure, which makes it easier to interact with water molecules, which is consistent with the high solubility of SPH–SL−30. This may be because a short period of hydrolysis disrupts soy protein molecular chains, exposing intramolecular hydrogen bonds and forming interpeptide chain hydrogen bonds, resulting in a stretched and flexible protein structure. However, prolonged enzymatic hydrolysis instead promotes aggregation between the molecular chains of soy protein, resulting in a compact protein structure. In addition, the ratio of random coil structures of SPH decreased slightly with increasing enzymatic hydrolysis time. This may be due to the shift of random coil structures to β−turn structures of SPH–SL caused by the homogenization treatment in the preparation of protein powder [17].

### 3.7. The Surface Hydrophobicity of SPH−SL at Different Hydrolysis Times

**Figure 7:**
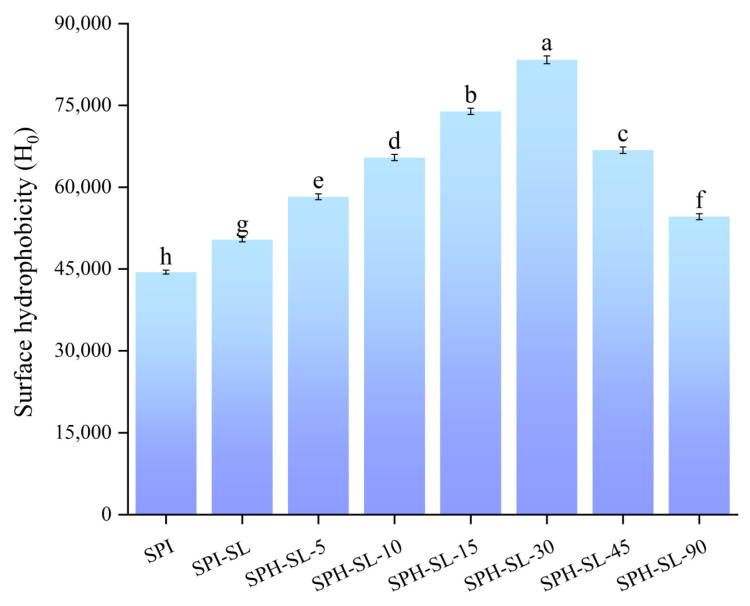
The surface hydrophobicity of SPH−SL at different hydrolysis times. The different superscript letters indicate significant differences (*p* < 0.05).

Surface hydrophobicity is related to the number of hydrophobic groups present on the surface of protein molecules, which is an important indicator for characterizing protein conformation and function [51]. Figure 7 illustrates the surface hydrophobicity of SPH−SL at different hydrolysis times. Figure 7 shows that the surface hydrophobicity of SPI−SL is higher than that of SPI. This may be because the hydrophobic interaction between SPI and SL changes the conformation of the proteins, thereby increasing the surface hydrophobicity of SPI−SL [52]. The surface hydrophobicity of SPH−SL increased and then decreased with increasing hydrolysis time, and the surface hydrophobicity of SPH–SL−30 was the highest. This may be related to the fact that short−term enzymatic hydrolysis fully stretches the structure of the complexes of soy protein−soy lecithin complexes, exposing hydrophobic sites within the molecule, resulting in the exposure of hydrophobic groups (e.g., tryptophan, tyrosine residues, and aliphatic hydrophobic groups) into the polar microenvironment [14]. Conversely, prolonged enzymatic hydrolysis may lead to the aggregation of some peptides through hydrophobic interactions or hydrogen bonding, etc., thus, reducing the number of hydrophobic groups [53]. In addition, the surface hydrophobicity of SPH–SL−30 is higher than that of SPI and SPH. This implies that the treatment of soy lecithin modification combined with Alcalase hydrolysis can significantly improve the surface hydrophobicity of soy protein powder. Amiri et al. [54] pointed out that most hydrophobic amino acids are negatively charged. Combined with zeta’s results, the high surface hydrophobicity of SPH–SL may indicate that more negatively charged hydrophobic amino acids are exposed, thereby leading to the increase in electrostatic repulsion between protein particles. Therefore, the increase in the hydrophobicity of SPH−SL and the increase in the zeta potential indicate that the improvement in solubility of SPH−SL may be related to the intermolecular interaction force.

### 3.8. The Bulk Density of SPH−SL at Different Hydrolysis Times

Bulk density is a crucial parameter in assessing the quality of protein powders, impacting factors such as transport costs, dosage control, fluidity, and solubility of protein flour [55]. This parameter is influenced by various factors including particle size, distribution, shape, and inter−particle friction. Table 2 illustrates the bulk density of SPH−SL at different hydrolysis times, with SPI exhibiting a bulk density of 0.2046 ± 001 Kg/m^3^. The bulk density of SPI−SL is 0.2095 ± 001 Kg/m^3^. The bulk density of SPH−SL increases and then decreases with increasing hydrolysis time. SPH–SL has a higher bulk density than SPI and SPI−SL, and, in particular, SPH−SL−30 has the highest bulk density. The higher bulk density values exhibited by SPH−SL are attributed to their smaller particle size and more uniform morphological appearance. This allows the soy protein to reach a compact packing state, thereby increasing the bulk density [56]. The higher packing bulk of SPH−SL prevents agglomeration and oxidation between protein particles, thereby enhancing their solubility [22]. In addition, the higher bulk density of SPH−SL indicates that the higher the quality of soy protein powder per unit volume, the better the product quality. In conclusion, compared with a single soy lecithin modification treatment, the treatment of soy lecithin modification combined with Alcalase hydrolysis can significantly increase bulk density and improve the quality of protein powder.

### 3.9. The Wettability of SPH−SL at Different Hydrolysis Times

The initial step in the rehydration process of powder involves wetting, which results in a transition from solid–gas to solid–liquid interfacial interactions [57]. The reduced wettability of soy protein powder may have significant implications for its utilization in food industrial and commercial settings. Table 2 displays the wetting times of soy protein hydrolysates at varying hydrolysis durations, with SPI demonstrating a wetting time of 7.06 ± 0.10 min. The wetting time of SPI−SL is shorter than that of SPI, potentially due to the exposure of the hydrophilic head of SL when combined with soy protein, thus promoting the rapid wetting of soy protein particles [58]. Alternatively, SL may play a role in facilitating the adsorption of soy protein at the water–air interface, thereby reducing interfacial tension and alleviating the inhibitory impact of powder sinking, ultimately leading to a decrease in wetting time [59]. SPH−SL exhibits a lower wetting time compared to SPI−SL. With increasing hydrolysis time, the wetting time of SPH−SL decreased first and then increased. SPH−SL−30 had the shortest wetting time of all samples. This may be due to the reduction in soy protein size and structural unfolding caused by short−term enzymatic hydrolysis, which promoted the complex of SL and soy protein, improved structural flexibility, and, thus, increased interaction with water. However, prolonged enzymatic hydrolysis may promote aggregation between soy proteins or soy peptide chains. This will lead to masking of binding sites with soy lecithin, reducing structural flexibility and preventing interactions with water.

As a result, compared with a single soy lecithin modification treatment, the treatment of soy lecithin modification combined with Alcalase hydrolysis can significantly increase the wettability, thereby improving the rehydration characteristics of soy protein powder.

### 3.10. The Dispersibility of SPH−SL at Different Hydrolysis Times

The dispersion mechanism of soy protein powder entails the dissolution of micron—sized powder clusters following wetting and immersion, leading to the liberation of nano—sized primary particles. Freudig et al. [60] suggested that only powders that have been sufficiently wetted and sunk into solution can be further dispersed. The dispersion time of SPH−SL at different hydrolysis times is shown in Table 2. The dispersion time of SPI is 31.28 ± 2.03 s. The dispersion time of SPI−SL is lower than that of SPI. SPH–SL exhibits a lower wetting time compared to SPI−SL. With increasing hydrolysis time, the dispersion time of SPH−SL decreased first and then increased. SPH–SL−30 had the shortest dispersion time of all samples. The dispersibility exhibited by all of the above samples is consistent with the trend in product wettability. Moreover, the pronounced electrostatic repulsion exhibited by SPH−SL facilitates the prevention of powder agglomeration at the air–water interface, thereby promoting the swift and uniform dispersion of the protein powder [61].

### 3.11. The Solubility of SPH−SL at Different Hydrolysis Times

The process of solubilization, occurring during the rehydration of powder, is a crucial stage in which primary particles begin to release materials from the surface of the particle into the liquid medium [62]. Enhancing the solubility of soy protein is essential for enabling the functional expression and production of soy protein. The solubility of soy proteins is influenced by factors such as electrostatic repulsion and the presence of hydrophilic groups on the protein surface [63]. The solubility of SPH−SL at various hydrolysis durations is presented in Table 2. Analysis of Table 2 reveals that SPI−SL exhibits superior solubility compared to SPI, possibly attributed to the incorporation of SL into soy protein, resulting in enhanced surface charge and improved interactions between protein and water molecules, leading to the formation of a hydration layer and soluble aggregates [64]. The solubility of SPH−SL initially increased with hydrolysis time before decreasing. Notably, SPH–SL−30 demonstrated the highest solubility among all tested samples. The enhanced solubility of SPH−SL following the short−term enzymatic hydrolysis may be attributed to the continuous cleavage of peptide bonds during alkaline protease hydrolysis, resulting in a decrease in the size of soy protein particles and thereby facilitating improved protein–water interaction. Meanwhile, after the short−term enzymatic hydrolysis, the spatial configuration of SPH−SL exhibited a gradual loosening, leading to an increase in electrostatic repulsion between proteins. This phenomenon facilitates the unfolding of soy proteins, diminishes aggregation among them, and enhances their interaction with water. These findings are consistent with the outcomes obtained from particle size analysis, scanning electron microscopy (SEM), zeta potential measurements, and Fourier transform infrared (FTIR) spectroscopy. Furthermore, following brief enzymatic hydrolysis, soy proteins may experience an increase in binding sites, facilitating the interaction between SL and soy proteins and enhancing the presence of hydrophilic groups on the protein surface, thereby enhancing solubility. Conversely, prolonged enzymatic hydrolysis may lead to the formation of soy protein aggregates, resulting in an enlargement of soy protein particles. This could potentially decrease the number of ionizable side−chain moieties exposed by SPH−SL and impede protein–water interactions. Furthermore, the formation of soy protein aggregates can obscure the binding sites with soy lecithin, resulting in a reduction in hydrophilic groups on the protein surface and subsequently decreasing the solubility of SPH−SL. Consequently, the combination of soy lecithin modification and Alcalase hydrolysis treatment proved to enhance the solubility of soy protein powder more effectively than the singular soy lecithin modification treatment. This improvement can be attributed to alterations in particle size, structural flexibility, and surface characteristics of soy protein.

### 3.12. The Viscosity of SPH−SL at Different Hydrolysis Times

**Figure 8:**
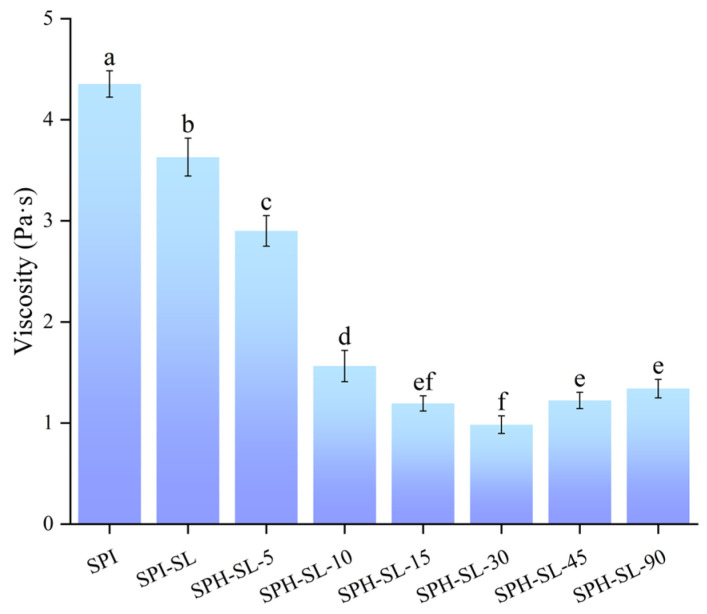
The viscosity of SPH–SL at different hydrolysis times. The different superscript letters indicate significant differences (*p* < 0.05).

The viscosity of soy protein solutions plays a crucial role in influencing the processing of food systems and the functional properties of proteins [65]. The viscosity of SPH−SL at different hydrolysis times is shown in Figure 8. Through Figure 8, it can be found that SPI–SL has a lower viscosity than SPI. This may be related to the fact that soy protein complexes with SL increase electrostatic repulsion and, thus, reduce particle size [66]. The viscosity of SPH−SL decreased and then increased with increasing hydrolysis time. SPH−SL−30 had the lowest viscosity among all the samples. The reduction in viscosity of soy protein hydrolysate−soy lecithin (SPH−SL) solutions may be attributed to enzymatic hydrolysis leading to a decrease in soy protein particle size, enhanced electrostatic repulsion between soy proteins, prevention of soy protein cross−linking, and ultimately a decrease in the apparent volume of soy protein [67]. However, prolonged enzymatic hydrolysis may diminish the electrostatic repulsion between soy proteins, facilitate the cross−linking of soy proteins, and enhance the apparent volume of SPH−SL [68] Overall, the lower viscosity of SPH−–SL means that SPH−SL has good processing properties (e.g., it is less prone to scaling in food transportation systems) [68]. In addition, the low viscosity of SPH–SL is more favorable for the processing of plant−based beverages [6]. Consequently, the combination of soy lecithin modification with Alcalase hydrolysis treatment notably decreases the viscosity of the soy protein solution in comparison to solely employing soy lecithin modification, thereby enhancing the processing characteristics of the soy protein powder.

### 3.13. The EAI and ESI of SPH−SL at Different Hydrolysis Times

**Figure 9:**
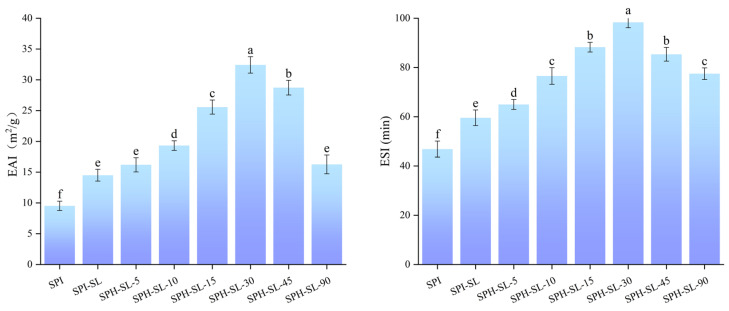
The EAI and ESI of SPH−SL at different hydrolysis times. The different superscript letters indicate significant differences (*p* < 0.05).

Emulsification is a crucial functional attribute of soy protein, particularly in the manufacturing of plant−based beverages and ice cream. Figure 9 illustrates the EAI and ESI values of SPH−SL at various hydrolysis durations. Analysis of the data reveals that the EAI and ESI values exhibit comparable trends across different hydrolysis times. The higher EAI and ESI values of SPI−SL compared to SPI can be attributed to the interaction between lecithin and soy protein, resulting in structural and interface modifications that enhance its emulsifying capacity [69]. The EAI and ESI of SPH−SL initially increased and then decreased as hydrolysis time increased. SPH–SL−30 exhibited the highest EAI and ESI among all samples. The improved emulsifying property of SPH−SL may be related to the fact that short−term enzymatic hydrolysis promotes the gradual exposure of hydrophobic groups in the protein fraction and its enhanced interaction with phospholipids, which results in faster adsorption of proteins onto the oil−water interface [48]. However, prolonged enzymatic hydrolysis may produce a large number of small molecule peptides, which are difficult to unfold at the interface and reduce the interfacial tension, leading to a decrease in the emulsifying properties of SPH−SL. In addition, the small molecular peptides do not readily interact with phospholipids, which may also account for the reduced emulsification performance of SPH−SL. In addition, the weak interaction of small molecular peptides with phospholipids may also contribute to the reduced emulsification stability of SPH−SL emulsions. In conclusion, the treatment of soy lecithin modification combined with Alcalase hydrolysis significantly improved the emulsification of soy protein solutions compared to single soy lecithin modification treatment, thereby improving the processing properties of the soy protein powder.

## 4. Conclusions

Soy protein powders with poor rehydration properties and low functionality severely limit the efficient utilization of raw materials and production of plant−based foods. The present study finds a treatment of soy lecithin modification combined with Alcalase hydrolysis improves soy protein powders’ rehydration and functional properties. This can contribute to the sustainable development of the plant−based food industry. SPH−SL powders were obtained by fixing the concentration of lecithin and treating it for different enzymatic times. Processing properties such as rehydration, viscosity, and emulsification of SPH−SL powders were investigated. Compared with SPI and SPI−SL, SPH–SL has smaller particle sizes, a uniform microstructure, high electrostatic repulsion, and high surface hydrophobicity. The FTIR results point out that SPH−SL has a looser structure that contributes to its solubility expression. These properties result in SPH–SL exhibiting high rehydration, low viscosity, and high emulsification characteristics, especially SPH–SL−30. In summary, the treatment of soy lecithin modification combined with Alcalase hydrolysis can effectively improve a series of functional properties of commercial soy protein powders, thus enabling their better application in the field of plant−based foods.

## Figures and Tables

**Table 1:** The secondary structure content of SPH−SL at different hydrolysis times.

Sample	α−Helix (%)	β−Sheet (%)	β−Turn (%)	Random Coil (%)	α−Helix/β−Sheet (%)
SPI	21.18 ± 0.23 ^bc^	38.89 ± 0.22 ^f^	23.65 ± 0.12 ^b^	17.28 ± 0.54 ^cd^	54.46 ± 0.28 ^b^
SPI−SL	20.85 ± 0.22 ^c^	39.67 ± 0.25 ^e^	19.86 ± 0.20 ^e^	19.59 ± 0.29 ^a^	52.56 ± 0.21 ^c^
SPH−SL−5	20.29 ± 0.23 ^d^	40.88 ± 0.18 ^d^	20.95 ± 0.21 ^d^	17.88 ± 0.38 ^b^	49.64 ± 0.70 ^d^
SPH−SL−10	19.94 ± 0.20 ^d^	41.28 ± 0.14 ^c^	21.22 ± 0.21 ^cd^	17.57 ± 0.13 ^bc^	48.30 ± 0.33 ^e^
SPH−SL−15	19.32 ± 0.20 ^e^	42.44 ± 0.24 ^b^	21.44 ± 0.22 ^c^	16.80 ± 0.19 ^d^	45.54 ± 0.72 ^f^
SPH−SL−30	18.52 ± 0.24 ^f^	43.40 ± 0.23 ^a^	22.44 ± 0.28 ^b^	15.65 ± 0.19 ^e^	42.67 ± 0.35 ^g^
SPH−SL−45	21.44 ± 0.20 ^b^	39.45 ± 0.22 ^e^	23.82 ± 0.15 ^a^	15.29 ± 0.36 ^e^	54.35 ± 0.27 ^b^
SPH−SL−90	22.83 ± 0.30 ^a^	37.95 ± 0.20 ^g^	23.99 ± 0.22 ^a^	15.23 ± 0.28 ^e^	60.16 ± 0.45 ^a^

Means with the different superscript letters within the same column for each parameter are significantly different (*p* < 0.05).

**Table 2:** The bulk density, wettability, dispersibility, and solubility of SPH–SL at different hydrolysis times.

Sample	Bulk Density (Kg/m^3^)	Wettability (min)	Dispersibility (s)	Solubility (%)
SPI	0.2046 ± 0.0007 ^g^	7.06 ± 0.10 ^a^	31.28 ± 2.03 ^a^	54.15 ± 0.60 ^f^
SPI−SL	0.2095 ± 0.0013 ^f^	6.22 ± 0.06 ^b^	26.12 ± 1.92 ^b^	57.94 ± 0.23 ^e^
SPH−SL−5	0.2137 ± 0.0015 ^e^	4.04 ± 0.05 ^d^	23.69 ± 1.98 ^b^	63.19 ± 0.57 ^d^
SPH−SL−10	0.2285 ± 0.0020 ^c^	3.29 ± 0.04 ^e^	19.29 ± 1.52 ^c^	71.70 ± 1.36 ^c^
SPH−SL−15	0.2318 ± 0.0028 ^bc^	3.24 ± 0.03 ^e^	13.51 ± 1.35 ^d^	85.30 ± 0.65 ^b^
SPH−SL−30	0.2629 ± 0.0040 ^a^	3.04 ± 0.02 ^f^	12.29 ± 1.58 ^d^	93.17 ± 0.74 ^a^
SPH−SL−45	0.2331 ± 0.0029 ^b^	3.26 ± 0.05 ^e^	18.17 ± 1.57 ^c^	86.22 ± 0.27 ^b^
SPH−SL−90	0.2194 ± 0.0015 ^d^	5.19 ± 0.07 ^c^	20.25 ± 1.75 ^c^	71.08 ± 1.00 ^c^

Means with the different superscript letters within the same column for each parameter are significantly different (*p* < 0.05).

## Data Availability

The original contributions presented in the study are included in the article, further inquiries can be directed to the corresponding author.
